# Centipede envenomation (Chilopoda): Case report

**DOI:** 10.1590/0037-8682-0601-2021

**Published:** 2022-06-06

**Authors:** Vidal Haddad, Paulo Cezar Haddad de Amorim, Carolina Rassi da Cruz, Antônio Lucas Sforcin Amaral

**Affiliations:** 1 Universidade Estadual Paulista, Faculdade de Medicina de Botucatu, Disciplina de Dermatologia, Botucatu, SP, Brasil.; 2 Universidade Estadual Paulista, Instituto de Biociências, Botucatu, SP, Brasil.

**Keywords:** Chilopoda, Bites and stings, Centipedes envenomation

## Abstract

Centipedes are venomous arthropods that have an elongated body, divided into many segments, presenting a pair of legs in each segment, adapted pairs of paws that simulate fangs and inject venom causing intense pain, with local erythema and edema, and rarely, blisters and skin necrosis. We present the case of a young woman pricked on her upper lip with intense swelling and local pain and discuss the real danger of envenomation and the therapeutic measures that should be taken.

## INTRODUCTION

**FIGURE 1:**
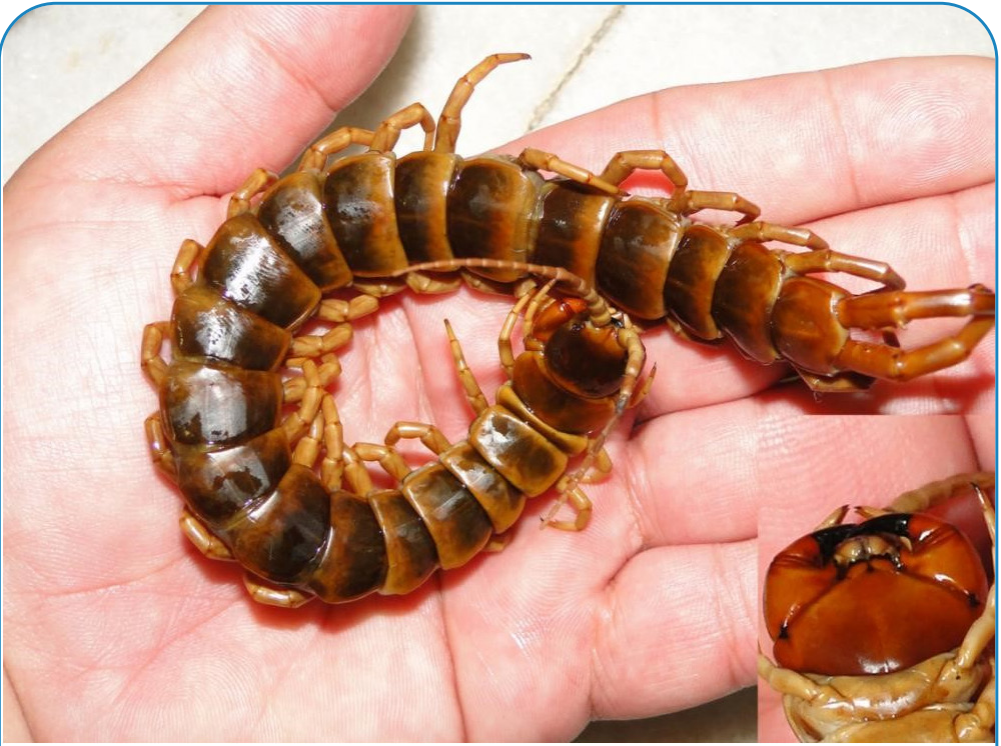
Centipede (*Scolopendra* sp.)

Centipedes are venomous arthropods of the Myriapod subphylum and Chilopoda classes, which have an elongated body divided into many segments, presenting a pair of legs in each segment (Figure 1). Approximately 8,000 species of centipedes live in humid environments, hiding in the ground and under tree barks^1^.

They are very effective predators, hunting vertebrates such as birds, snakes, bats, and amphibians, but prefer cockroaches and other invertebrates, which cause 

them to occasionally enter houses through sewers. They are agile, unlike the millipede (Diplopoda) with which they are confused^1^ ^),(^ ^2^.

Its forcipules are adapted pairs of paws that simulate fangs (Figure 1) and inject venom, which causes intense pain, local erythema and edema, and rarely, blisters and skin necrosis. In some cases, headaches, fever, malaise, anxiety, and dizziness were also observed. Envenomation is not described as potentially fatal, even in children, but these animals can bite repeatedly, which increases pain^3^ ^),(^ ^4^ ^),(^ ^5^ ^),(^ ^6^. Rare reports of human deaths are unconvincing, and secondary infection is the main complication of injury^6^. Almost all envenomations caused by centipedes resolve spontaneously without complications. The bite site should be washed with soap, and water and cold compresses are useful. Systemic analgesics are recommended for pain management.

The forcipules are responsible for the injection of the venom.

The venom of the centípedes contains histamine, serotonin, and toxins with cardiotoxic, myotoxic, and neurotoxic effects. Rarely, bites can cause systemic effects, and clinical reports point to the possibility of severe allergic reactions, especially in victims with allergies to bee and wasp stings^7^.

In Brazil, the most common species are *Scolopendra viridicornis* and *S. subspinipes*, which can reach up to 12 cm in diameter, especially *S. viridicornis* ^1^.

## CASE REPORT

A 21-year-old female patient was asleep and felt a strong burning pain in her upper lip in the middle of the night. As the room was dark, her reaction was to put her hand to her mouth, and when pulling something hanging from her lip, she felt that it was firmly adhered, taking a few seconds to be removed.

**FIGURE 2:**
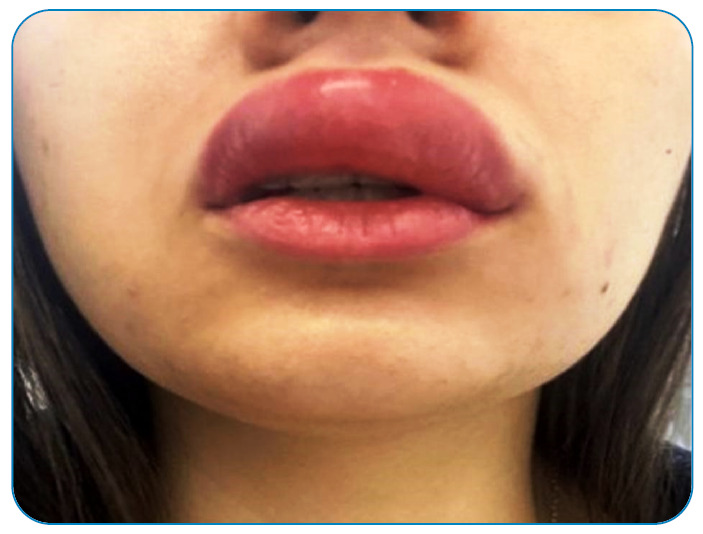
Intense edema in the upper lip of the patient caused by the bite of the centipede.

When the patient turned on the light, already feeling intense pain at the site of the bite, she saw that her lip had a small cut and slight bleeding when she noticed a large centipede (approximately 10 cm) on the cover (Figure 2).

**FIGURE 3:**
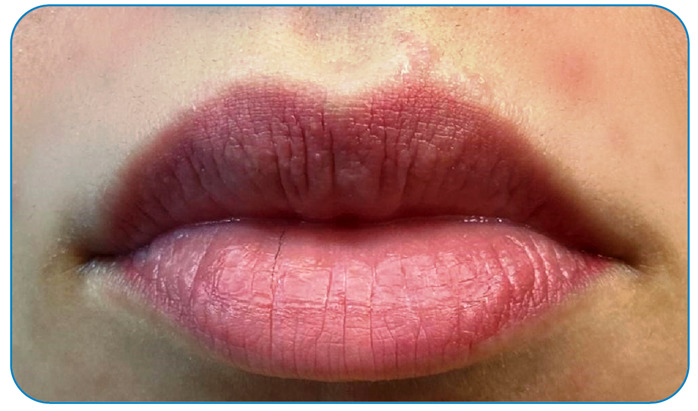
Regression of the edema after 2 days and small marks of the bite in the patient’s upper lip.

As the pain increased in intensity, the patient was taken to an emergency care center, where the captured animal was identified as a centipede, without registration of the species. The patient felt her lip swell, throat hurt, and shortness of breath (Figure 3). The patient was treated with antihistamines (promethazine) and injectable analgesics (dipyrone) and reassured about the intensity of the envenomation, remained under observation for 2 h, and was released after pain control. The edema subsided within approximately 2 days.

## DISCUSSION

Reported envenomation causes pain, erythema, and edema. In this case, the loose connective tissue in the region showed more intense edema. Despite being intense, the pain subsided with injectable analgesics. Antihistamines were applied because of the upper lip edema (a clinical condition similar to allergic angioedema).

Pain control decisively contributed to envenomation treatment, as the observed breathing difficulties may have been provoked or aggravated by the stress caused by urgency and pain. Centipede bites are part of the popular imagination as serious and potentially fatal envenomations, but this is not the reality observed in the reported cases.

### Ethical Approval

The report of one case is not required to be submitted to the Ethical Approval of the CEP of Faculdade de Medicina de Botucatu.
